# A hands-on tutorial on network and topological neuroscience

**DOI:** 10.1007/s00429-021-02435-0

**Published:** 2022-02-10

**Authors:** Eduarda Gervini Zampieri Centeno, Giulia Moreni, Chris Vriend, Linda Douw, Fernando Antônio Nóbrega Santos

**Affiliations:** 1 Amsterdam Neuroscience, Vrije Universiteit Amsterdam, Anatomy and Neurosciences, Amsterdam UMC, De Boelelaan 1117, Amsterdam, The Netherlands; 2 Institut Des Maladies Neurodégénératives, UMR 5293, Université de Bordeaux, CNRS, Bordeaux Neurocampus, 146 Rue Léo Saignat, 33000 Bordeaux, France; 3 Amsterdam Neuroscience, Vrije Universiteit Amsterdam, Psychiatry, Amsterdam UMC, De Boelelaan 1117, Amsterdam, The Netherlands; 4 Institute for Advanced Studies, University of Amsterdam, Oude Turfmarkt 147, 1012 GC Amsterdam, The Netherlands

**Keywords:** Network analysis, Neuroscience, Topological data analysis, Python, Graph theory, Brain networks

## Abstract

The brain is an extraordinarily complex system that facilitates the optimal integration of information from different regions to execute its functions. With the recent advances in technology, researchers can now collect enormous amounts of data from the brain using neuroimaging at different scales and from numerous modalities. With that comes the need for sophisticated tools for analysis. The field of network neuroscience has been trying to tackle these challenges, and graph theory has been one of its essential branches through the investigation of brain networks. Recently, topological data analysis has gained more attention as an alternative framework by providing a set of metrics that go beyond pairwise connections and offer improved robustness against noise. In this hands-on tutorial, our goal is to provide the computational tools to explore neuroimaging data using these frameworks and to facilitate their accessibility, data visualisation, and comprehension for newcomers to the field. We will start by giving a concise (and by no means complete) overview of the field to introduce the two frameworks and then explain how to compute both well-established and newer metrics on resting-state functional magnetic resonance imaging. We use an open-source language (Python) and provide an accompanying publicly available Jupyter Notebook that uses the 1000 Functional Connectomes Project dataset. Moreover, we would like to highlight one part of our notebook dedicated to the realistic visualisation of high order interactions in brain networks. This pipeline provides three-dimensional (3-D) plots of pairwise and higher-order interactions projected in a brain atlas, a new feature tailor-made for network neuroscience.

**Supplementary Information:**

The online version contains supplementary material available at 10.1007/s00429-021-02435-0.

## Introduction

Neuroscience is still a young research field, with its emergence as a formal discipline happening only around 70 years ago (Cowan et al. 2000). The field has since mushroomed, and much of our current knowledge about the human brain’s neurobiology was made possible by the rapid advances in technologies to investigate the brain in vivo at high-resolution and different scales. An example is magnetic resonance imaging (MRI), which allows us to measure regional characteristics of the brain’s structure non-invasively and may also be used to assess anatomical and functional interactions between brain regions (Rosen and Savoy 2012; Sizemore et al. 2018). This expansion in the field led to an exponential increase in data size and complexity. To analyse and interpret this ‘big data’, researchers had to develop robust theoretical frameworks. Complex network science was brought to neuroscience and has been increasingly used to study the brain’s intricate communication and wiring (Bassett and Sporns 2017; Sporns 2018). The resulting field—network neuroscience—aims to see the brain through an integrative lens by mapping and modelling its elements and interactions (Bassett and Sporns 2017; Fornito et al. 2016).

One of the main theoretical frameworks from complex network science used to model, estimate, and simulate brain networks is graph theory (Gross and Yellen 2003; Bullmore and Sporns 2009). A graph is comprised of a set of interconnected elements, also known as *vertices* and *edges*. Vertices (also known as nodes) in a network can, for example, be brain areas, while edges (also known as links) are a representation of the functional connectivity between pairs of vertices (Sporns 2018). Various imaging modalities are available to reconstruct the brain network (Hart et al. 2016; Bullmore and Sporns 2009). The focus of this hands-on paper will be resting-state functional MRI (rsfMRI). As the name suggests, rsfMRI indirectly measures brain activity while a subject is at rest (i.e., does not perform any task). This type of data provides information about spontaneous brain functional connectivity (Raichle 2011). Functional connectivity is often operationalised by a statistical dependency (usually a Pearson correlation coefficient) between signals measured from anatomically separated brain areas (Rosen and Savoy 2012; Smith et al. 2013). An in-depth explanation of rsfMRI and functional connectivity is out of the scope of our manuscript. However, considering the focus on this type of data here, we recommend readers who are not familiar with this imaging method to read Lee et al. (2013); van den Heuvel and Hulshoff Pol (2010); Smith et al. (2013); Smitha et al. (2017) for a comprehensive overview.

Several descriptive graph metrics^1^ (Do Carmo 2016) can be calculated to describe the brain network’s characteristic; examples include the degree or the total number of connections of a vertex and the path length (number of intermediate edges) between two vertices (Fornito et al. 2016; Hallquist and Hillary 2018). These metrics have consistently allowed researchers to identify non-random features of brain networks. A key example is the ground-breaking discovery that the brain (like most other real-world networks) follows a ‘small-world network’ architecture (Bassett and Bullmore 2017; Bassett and Sporns 2017; Watts and Strogatz 1998). This refers to the phenomenon that, to minimise wiring cost while simultaneously maintaining optimal efficiency and robustness against perturbation, the brain network obeys a balance between the ability to perform local processing (i.e., segregation) and combining information streams on a global level (i.e., integration).

Network neuroscience has thereby offered a comprehensive set of analytical tools to study not only the local properties of brain areas but also their significance for the entire brain network functioning. Using graph theory, many insights have been gathered on the healthy and diseased brain neurobiology (Farahani et al. 2019; Hallquist and Hillary 2018; Hart et al. 2016; Sporns 2018).

Another perspective on the characteristics of the brain network can be provided by (algebraic) topological data analysis (TDA), by analysing the interactions between a set of vertices beyond the ‘simple’ pairwise connections (i.e., higher-order interactions). With TDA, one can identify a network’s ‘shape’ and its invariant properties [i.e., coordinate and deformation invariances (Zomorodian 2005; Offroy and Duponchel 2016)]. Thus, as we will illustrate along with the manuscript, TDA often provides more robustness against noise than graph theoretical analysis (Blevins and Bassett 2020; Blevins et al. 2021), which can be a significant issue in imaging data (Sizemore et al. 2019; Liu 2016; Greve et al. 2013). Although TDA has only recently been adopted in network neuroscience (Curto and Itskov 2008; Singh et al. 2008), it has already shown exciting results on rsfMRI (Expert et al. 2019; Curto 2017). For example, group-level differences in network topology have been identified between healthy subjects that ingested psilocybin (psychedelic substance) and the placebo group (Petri et al. 2014) and between attention-deficit/hyperactivity disorder children and typically developing controls (Gracia-Tabuenca et al. 2020). A limitation of this framework is that the complexity and level of mathematical abstraction necessary to apply TDA and interpret the results might keep clinical neuroscientists without prior mathematical training from using it. Moreover, the high-order interaction structure that emerges from TDA analysis is often challenging to visualise realistically and understandably. Despite technical constraints, TDA allows us to deal with high order and large combinatorial coding capacity properly.

Therefore, we would like to facilitate the use of network neuroscience and its constituents graph theory and TDA by the general neuroscientific community by providing a step-by-step tutorial on how to compute different metrics commonly used to study brain networks and realistic high-order network plots. We offer a theoretical and experimental background of these metrics and include code blocks in each section to explain how to compute the different metrics. We also list several additional resources (Tables 1 and 2) of personal preference (and by no means complete), including a Jupyter Notebook that we created to accompany this hands-on tutorial publicly available on GitHub and Zenodo (Centeno and Santos 2021) (see Table 1, under the Jupyter Notebooks section—Notebook for network and topological analysis in neuroscience).

**Table 1:** List of computational resources

Name	Brief explanation	Source
Jupyter Notebooks		
AML-days-TDA-tutorial	A set of notebooks on the theory and applications of TDA pipelines	https://github.com/lordgrilo/AML-days-TDA-tutorial
DyNeuSR	Notebook on how to use Mapper—an algorithm for high dimensional dataset exploration	https://github.com/braindynamicslab/dyneusr-notebooks/
Notebook for network and topological analysis in neuroscience	Notebook on how to compute both classical and newer metrics of network and topological neuroscience	https://github.com/multinetlab-amsterdam/network_TDA_tutorial
NI-edu	A collection of neuroimaging-related course materials developed at the University of Amsterdam covering fMRI basic concepts and methodology	https://github.com/lukassnoek/NI-edu
Tutorials for Topological Data Analysis with the Gudhi Library	A collection of notebooks for the practice TDA with the Python Gudhi library	https://github.com/GUDHI/TDA-tutorial/
MATLAB toolboxes and scripts		
CliqueTop	A collection of MATLAB scripts for TDA	https://github.com/nebneuron/clique-top
The brain connectivity toolbox	MATLAB toolbox for brain network analysis	https://sites.google.com/site/bctnet/
Python packages and scripts		
Data visualisation		
DyNeuSR	“DyNeuSR is a Python visualisation library for topological representations of neuroimaging data.”	https://braindynamicslab.github.io/dyneusr/
Nxviz	“*nxviz* is a graph visualisation package for NetworkX.”	https://nxviz.readthedocs.io/
Plotly	“Plotly’s Python graphing library makes interactive, publication-quality graphs.”	https://plot.ly/python/
Graph theory		
Bctpy	“A direct translation to Python of the MATLAB brain connectivity toolbox.”	https://github.com/aestrivex/bctpy
NetworkX	“A package for the creation, manipulation, and study of the structure, dynamics, and functions of complex networks.”	https://networkx.github.io/
TDA		
Dionysus	“A library for computing persistent homology. It is written in C + + , with Python bindings.”	https://mrzv.org/software/dionysus2/
Giotto	“A collection of algorithms that harbours theoretical and technological advances spanning several key disciplines, including TDA.”	https://giotto.ai/
Gudhi	“The library offers state-of-the-art data structures and algorithms to construct simplicial complexes and compute persistent homology.”	http://gudhi.gforge.inria.fr/
Scikit-TDA	“Topological Data Analysis Python libraries intended for non-topologists”.	https://scikit-tda.org/
Topology ToolKit	“The Topology ToolKit (TTK) is an open-source library and software collection for topological data analysis and visualisation. Written in C + + but comes with Python bindings”.	https://topology-tool-kit.github.io/index.html

Table content was organised in alphabetic order

**Table 2:** List of reading resources

Name	Source
Key articles and books	
Cliques and cavities in the human connectome	Sizemore et al. (2018)
Network neuroscience	Bassett and Sporns (2017)
Fundamentals of brain network analysis (*the primary reference of our hands-on tutorial*)	Fornito et al. (2016)
Graph theory approaches to functional network organisation in brain disorders: a critique for a brave new small-world	Hallquist and Hillary (2018)
Topology for computing	Zomorodian (2005)
The importance of the whole: Topological data analysis for the network neuroscientist	Sizemore et al. (2019)
Editorial: Topological Neuroscience	Expert et al. (2019)
What can topology tell us about the neural code?	Curto (2017)
Homological scaffolds of brain functional networks	Petri et al. (2014)
Two’s company, three (or more) is a simplex	Giusti et al. (2016)
A roadmap for the computation of persistent homology	Otter et al. (2017)
Networks beyond pairwise interactions: structure and dynamics	Battiston et al. (2020)
Clique topology reveals intrinsic geometric structure in neural correlations	Giusti et al. (2015)
Computational topology: an introduction	Edelsbrunner and Harer (2010)

Table content was organised in numerical order

Our work differs from previous literature (Hallquist and Hillary 2018; Otter et al. 2017) since we describe the concepts central to graph theory and TDA and provide an easy-to-grasp step-by-step tutorial on how to compute these metrics using an easily accessible, open-source computer language. Furthermore, we offer new 3-D visualisations of simplicial complexes and TDA metrics in the brain that may facilitate the application and interpretation of these tools. Finally, we would like to stress that even though this tutorial focuses on rsfMRI, the main concepts and tools discussed in this paper can be extrapolated to other imaging modalities, biological or complex networks.

Since graph theory has been extensively translated for neuroscientists elsewhere, we refer the reader to the book in Fornito et al. (2016). This tutorial mainly focused on the topics covered in chapters 3, 4, 5, and the particular sections of chapters 6, 7, 8, and 9 about assortativity, shortest paths and the characteristic path length, the clustering coefficient, and modularity. In the second part of the tutorial, we explore hands-on TDA metrics, providing a summary of both theoretical and neuroscientific aspects with the calculations used in our work. We believe that our tutorial, which is far from being exhaustive, can make this emerging branch of network and topological neuroscience accessible to the reader. The codes we provide only require the knowlege of the functional connectivity matrix. For our realistic 3-D visualisation of simplicial complexes, we only need the coordinates of the nodes of a given brain atlas. Therefore, our scripts can be adapted to different databases, image modalities, and brain atlas. A short glossary with the key terms to understand this manuscript can be found in Table 3.

**Table 3:** Glossary with key terms

Term	Brief explanation
Clique complex	A simplicial complex constituted of all cliques of a network
Clique participation rank	The number of *k*-cliques in which a vertex $i$ participates for density *d*
Connectivity matrix	A square N x N matrix is used to represent connectivity between vertices
Face	A subset of a *k*-simplex. For example, if the *k*-simplex is a 2-simplex (triangle), all edges and vertices composing this simplex are also its faces
Filtration	A nested sequence of simplicial complexes
Functional magnetic resonance imaging (fMRI)	The imaging technique used to measure brain activity by detecting brain blood flow changes, i.e., blood-oxygen-level-dependent (BOLD) signal
*k*-clique	A subset of k vertices in an undirected graph in which all vertices are connected to each other
*k*-simplex	Geometrically, it is the generalisation of the region delimited by a tetrahedron to an arbitrary dimension k, which can be done in many ways (Zomorodian 2005). In this work, a k-simplex is a complete graph of k + 1 vertices. For example, 0-simplex is a point (or vertex), 1-simplex is a line segment (or edge), 2-simplex is a triangle, and so on
Simplicial complex	A simplicial complex K is a finite set of *k*-simplexes (e.g., vertices, edges, triangles, tetrahedrons, and their n-dimensional counterparts). The formal definition states that if K contains a *k*-simplex, then K also contains all faces of this *k*-simplex. Moreover, if two simplexes in K intersect, then this intersection is a face of each of them

Table content was organised in alphabetic order

## Hands-on tutorial

### General requirements

The following Python 3 packages are necessary to perform the computations presented below. The accompanying Jupyter Notebook can be found on GitHub (Table 1) or Zenodo (Centeno and Santos 2021).

### Code example


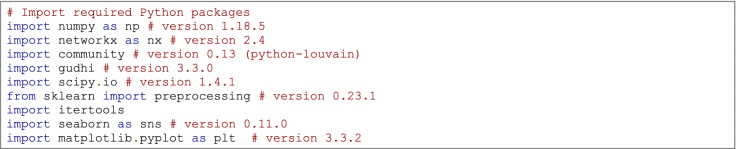


### The basis: the adjacency matrix

**Fig. 1:**
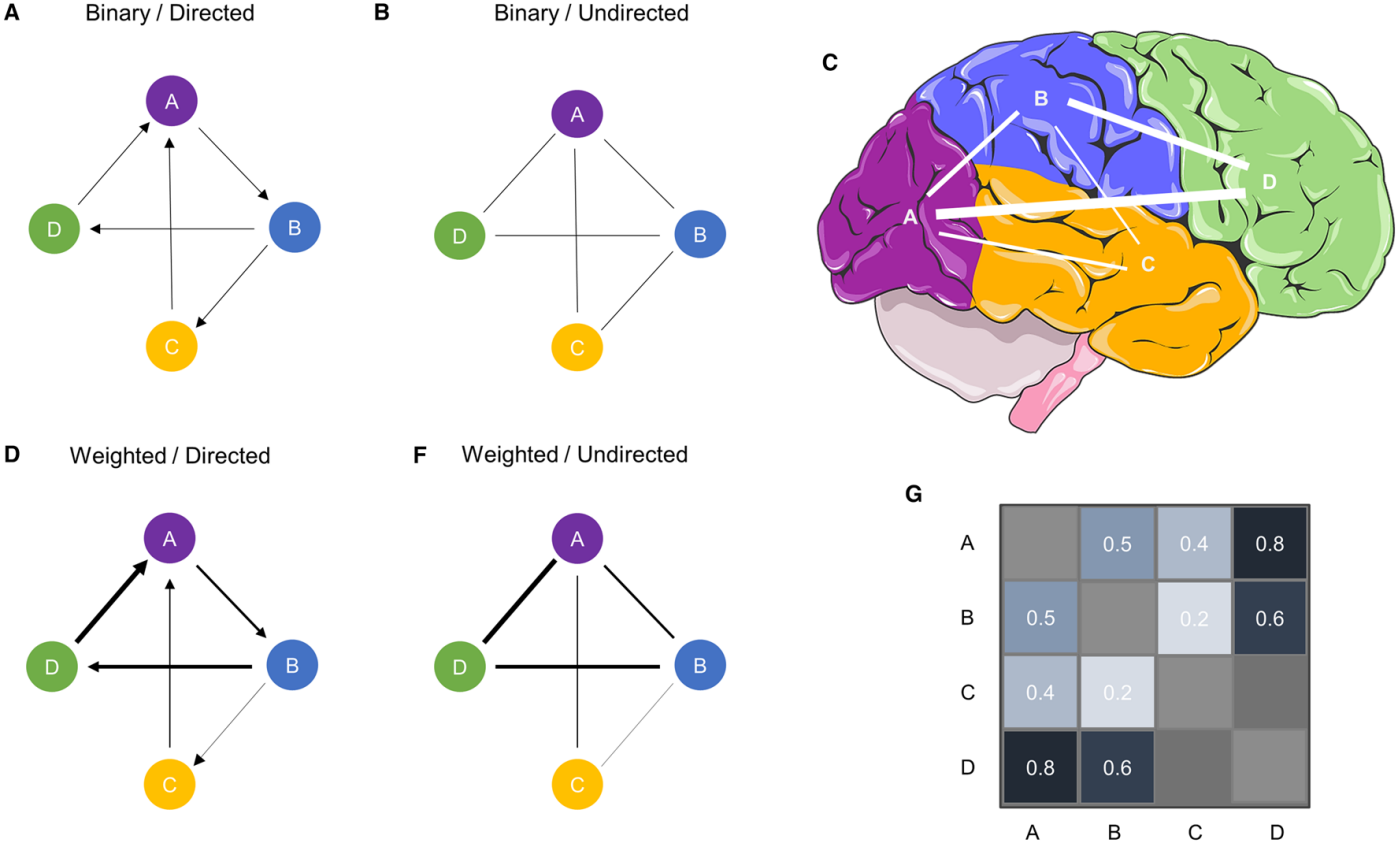
Types of networks. **a** A binary directed graph. **b** Binary, undirected graph. In binary graphs, the presence of a connection is signified by a 1 or 0 otherwise. **c** A representation of graph *f* as a network of brain areas. **d** A weighted, directed graph. **f** A weighted, undirected graph. In a weighted graph, the absolute strength of the connections is often represented by a number $w$, where $0\le w\le 1.$ **g** A connectivity matrix of *c* and *f*. Source: Part of the image was obtained from smart.servier.com

The basic unit on which graph theory and TDA are applied in the context of rsfMRI in our work is the adjacency or functional connectivity matrix (Fig. 1g), which presents the connections between all vertices in the network (Bassett and Sporns 2017; Fornito et al. 2016; Sporns 2018; Sporns et al. 2000). Typically, rsfMRI matrices are symmetric and do not specify the direction of connectivity (i.e., activity in area A drives activity in area B), thus yielding undirected networks (Fig. 1b and f). In contrast, non-symmetric matrixes would produce directed networks.

Before calculating any metrics on such matrices, several crucial factors must be considered when dealing with connectivity data (Jalili 2016; Hallquist and Hillary 2018). One critical decision is whether one wants to keep the information about edge weights. When the edges’ weights (e.g., correlation values in rsfMRI connectivity) are maintained, the network will be weighted (Fig. 1d and f). Another approach is to use an arbitrary threshold or density, e.g., only keep and binarise the 20% strongest connections (Fig. 1a and b). There is currently no gold standard for the weighting issue in rsfMRI matrices (Fornito et al. 2016; Jalili 2016) and may also be dependent on the dataset or proposed analysis (van den Heuvel et al. 2017). Brain network data are often analysed using a specific thresholding procedure (or criteria). These thresholded brain networks often display ubiquitous signatures of the brain as a complex system, such as skewed degree distributions, clustering, Giant components, small wordness, and short average path lengths, to name a few (Eguíluz et al. 2005). However, some of these properties, considered signatures of complex networks, are observed, even when one threshold normally distributed data (Cantwell et al. (2020). Yet, some brain network properties are not robust towards changes in the threshold (Garrison et al. 2015). Those results raised the awareness for using methods of network analysis that are independent of thresholding, such as minimum spanning trees (van Dellen et al. 2018; Stam et al. 2014) or topological data analysis (Phinyomark et al. 2017), as we will discuss below. Please see Chapter 11 in Fornito et al. (2016) and van Wijk et al. (2010); Simpson et al. (2013) for a more in-depth discussion on the issue of matrix thresholding and statistical connectomics.

Another relevant discussion about rsfMRI matrices is the interpretation of negative weights or anticorrelations. The debate of what such negative correlations mean in neurophysiology is still going on (Zhan et al. 2017). Studies have suggested that they could be considered artefacts introduced by global signal regression or pre-processing methods or simply by large phase differences in synchronised signals between brain areas (Chen et al. 2011; Murphy et al. 2009). Nevertheless, a few authors have suggested that anticorrelations might carry biological meaning underlying long-range synchronisation and that in diseased states, alterations in these negative correlations could indicate network reorganisation (Chen et al. 2011; Zhan et al. 2017). Negative weights can be absolutised to keep the potential biological information they may carry. If one decides to discard them, it is crucial to remember that some physiological information might be lost (Chen et al. 2011; Zhan et al. 2017; Fornito et al. 2016; Hallquist and Hillary 2018).

In this tutorial, we will use an undirected, absolutised (positively) weighted matrix. In our Jupyter notebook tutorial on GitHub (Centeno and Santos 2021), we provide an example matrix which is an average of all connectivity matrices available in our repository.

To follow the steps below, we assume that rsfMRI was already pre-processed and converted to a matrix according to some atlas. Steps and explanations on data pre-processing and atlas choices are beyond the scope of this paper; please see Strother (2006) or the NI-edu course materials (Table 1) for further information. Details on our Jupyter Notebook’s dataset pre-processing can be found in Brown et al. (2012); Biswal et al. (2010).

### Code example


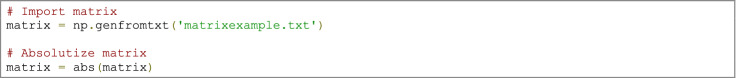


When working with fMRI brain network data, it is helpful to generate some plots (e.g., the heatmaps for matrix visualisation and distribution plots of edge weights) to facilitate data exploration, comprehension, and flag potential artefacts. In brain networks, we expect primarily weak edges and a smaller proportion of strong ones. When plotted as a probability density of log10, we expect the weight distribution to have a Gaussian-like form Fornito et al. (2016).

### Code example


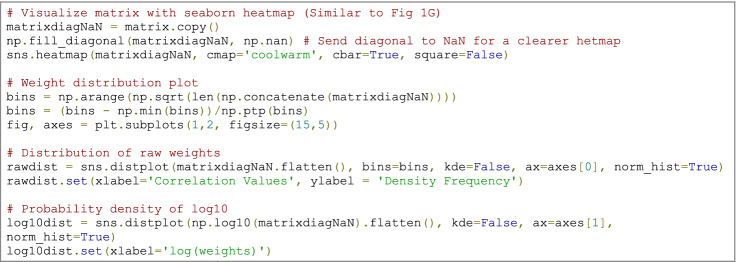


### Graph theory

**Fig. 2:**
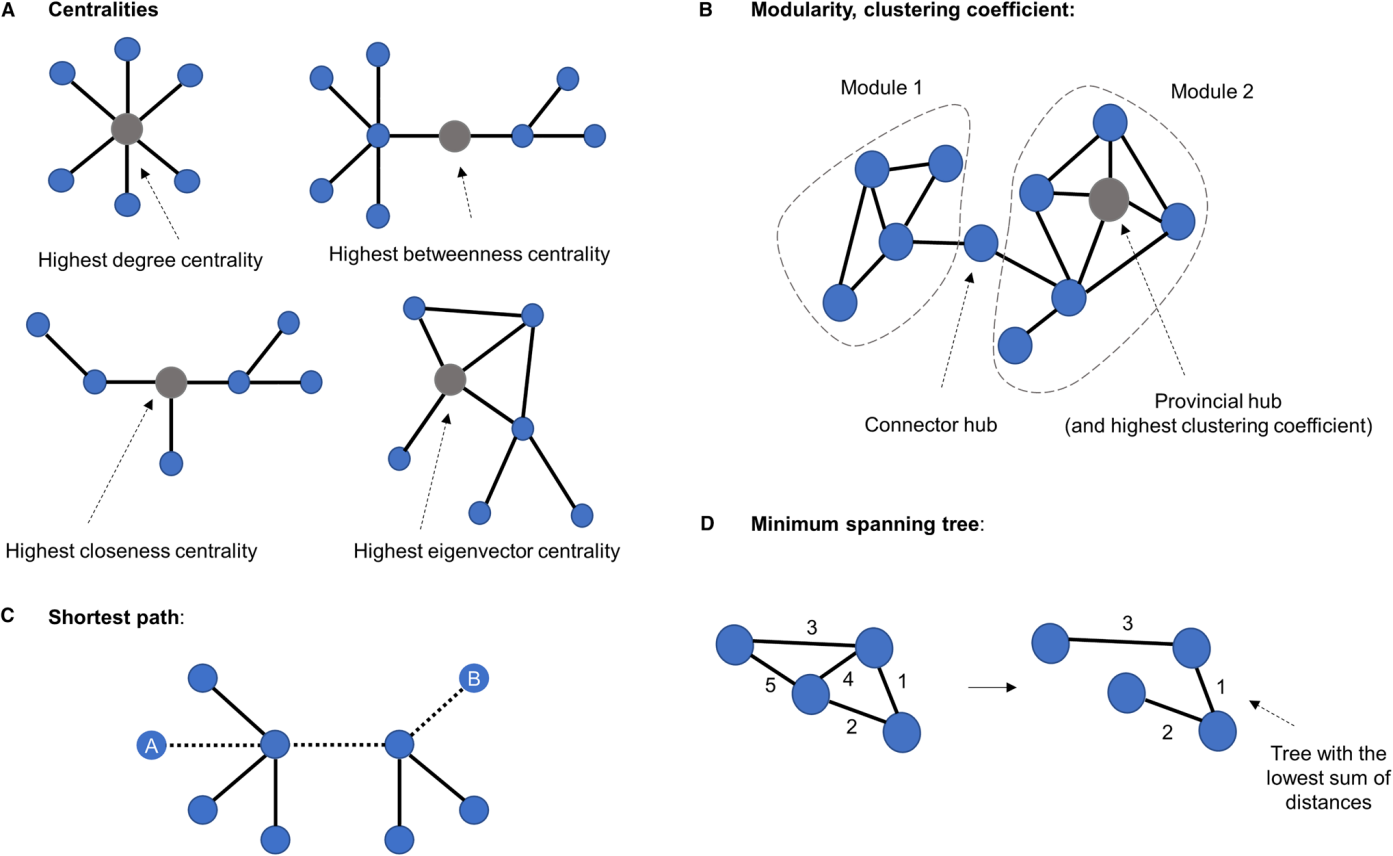
Graph theoretical metrics. **a** A representation of a graph indicating centralities. Highest degree centrality indicates the vertex with the most connections. Highest betweenness centrality refers to the vertex with most short paths passing through it. Highest closeness centrality denotes the vertex that needs the least edges to reach all the other nodes. The highest eigenvector centrality is achieved by the vertex best connected to the rest of the network, considering the number of neighbours and how well connected they are. **b** Representation of modularity and clustering coefficient. The latter indicates the tendency for any two neighbours of a vertex to be directly connected to each other. **c** The shortest path between vertices *a* and *b*. **d** The minimum spanning tree is a subset of a graph’s edges, which does not contain cycles, and that has the lowest sum of distances

Here, we will cover the most commonly used graph metrics in network neuroscience (see Fig. 2), also in line with Fornito et al. (2016). First, we need to create a graph object using the package *NetworkX* (Hagberg et al. 2008) and remove the self-loops (i.e., the connectivity matrix’s diagonal).

### Code example


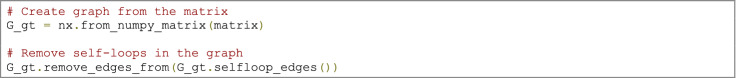


### Degree

Vertex *degree* quantifies the total number of vertex connections in an undirected binary network (Fornito et al. 2016). In an undirected weighted network like our rsfMRI matrix, the vertex degree is analogous to the vertex *strength* (i.e., the sum of all edges of a vertex) and equivalent to its degree centrality. This metric is one of the most fundamental metrics in network analysis and is a useful summary of how densely individual vertices are connected. It can be computed as the sum of edge weights of the neighbours of vertex $$i $$ as follows:

$${C}_{D} = {s}_{i } = \sum_{j\ne i}{w}_{\mathrm{ij}},$$

where ${w}_{ij}$ is the weight of the edge linking vertices $i$ and $j$.

### Code example


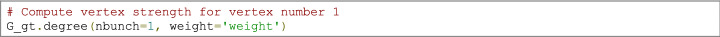


By removing the argument *weight* from the function, one can compute the degree of binarised networks where all edges are either 0 or 1 (useful if working with a sparse/not fully connected matrix). This change will give the vertex degree by calculating the number of edges adjacent to the vertex. One can also remove the specified vertex to estimate the degree/strength of all vertices. The *degree/strength distribution* allows us to scope the general network organisation in a single shot by displaying whether the network contains a few highly connected vertices, i.e., hubs (Hallquist and Hillary 2018).

### Path length

- The *shortest path* is the path with the least number of edges (or least total weight) between two vertices in a network. In a weighted graph, the shortest path is calculated by the minimum sum of the weights of edges between two vertices (Fornito et al. 2016). It is seen as a measure for understanding the efficiency of information diffusion in a network. Several algorithms can calculate path lengths, but Dijkstra’s algorithm (Dijkstra 1959) is one of the oldest and most well-known. An important detail is that this algorithm is only applicable to graphs with non-negative weights (Dijkstra 1959).
  A pivotal point to keep in mind is that in the case of correlation matrices, such as rsfMRI data, the weights must be converted to ‘distance’ by computing the inverse of the original weight ($1-weight$ or $\frac{1}{weight}$); a higher correlation value represents a shorter distance (Fornito et al. 2016). This conversion is essential for all the following path-based metrics.
- *Average path length *(*or characteristic path length*) is the average shortest path length for all possible pairs of vertices in a network. It is a global measure of information transport efficiency and integration in a network and is widely known due to the famous Watts–Strogatz model (Watts and Strogatz 1998). It can be computed as follows:
  $$\mathrm{L }= \sum_{i,j\in \mathrm{V}} \frac{d\left(i,j\right)}{N\left(N-1\right)},$$

where $\mathrm{V}$ is a set of vertices, $d\left(i,j\right)$ is the shortest path between vertices $i$ and $j$, and $N$ is the number of vertices in the network.

### Code example


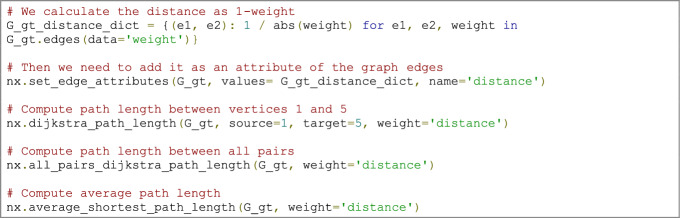


### The clustering coefficient

The clustering coefficient assesses the tendency for any two neighbours of a vertex to be directly connected (or more strongly connected in the weighted case) to each other and can also be termed cliquishness (Hallquist and Hillary 2018; Watts and Strogatz 1998). This metric is also used to compute the small-worldness coefficient (ratio between the characteristic path length and the clustering coefficient relative to random networks) (Watts and Strogatz 1998). The formula can be defined as follows:

$$\mathrm{Cl}=\frac{2}{{s}_{i}\left({s}_{i}-1\right)}\sum_{\mathrm{j},\mathrm{h}}{\left({\widehat{w}}_{ij}{\widehat{w}}_{jh}{\widehat{w}}_{hi}\right)}^\frac{1}{3},$$

where ${s}_{i}$ is the degree/strength of vertex$i$, and the edge weights are normalised by the maximum weight in the network, such that ${\widehat{w}}_{ij} =\frac{{w}_{ij}}{\mathrm{max}\left(w\right)}$.

### Code example


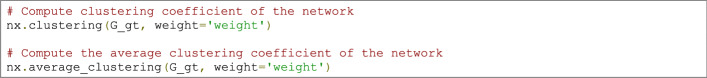


### Centralities

- *Eigenvector *(*degree-based*)* centrality* measures a vertex’s importance in a network while also considering its neighbours’ influence (Golbeck 2013). Thus, it considers both the quantity and quality of a vertex’s connections. The eigenvector centrality can be computed from the spectra of the adjacency matrix:
  $$Ax = \mathrm{\lambda x},$$
  where A is the adjacency matrix, and $x$ is an eigenvector of A with eigenvalue λ. We can now define the eigenvector centrality of a vertex $i$ as the following sum over its neighbours:
  $${C}_{E}\left(\mathrm{i}\right) = \frac{1}{{\uplambda }_{1}} \sum_{j=1}^{N} {A}_{ij}{x}_{j.}$$
  For weighted networks, certain conditions apply. According to the Perron–Frobenius theorem, the adjacency matrix’s largest eigenvalue, denoted here by ${\lambda }_{1}$, must be unique and positive, guaranteed only for matrices with positive values (Fornito et al. 2016; Newman 2008).
- *Closeness *(*shortest path-based*)* centrality* measures how closely or’ directly’ connected a vertex is to the rest of the network. If the vertex is the closest to every other element in the network, it has the potential to spread information fast and efficiently (Fornito et al. 2016). Formally, the closeness centrality of a vertex $i$ is the inverse of its average shortest path length (*N.B.* weights need to be converted to distances) to all N − 1 other vertices:
  $$ Cc(i) = \frac{N - 1}{{\sum\nolimits_{i = 1}^{N - 1} {d(i,\,j)} }}, $$
  where d($i$, $j$) is the shortest-path distance between $i$ and $j$, and $N$ is the number of vertices in the graph. In weighted networks, closeness centrality can be estimated by considering the summed weight of the shortest paths according to Dijkstra’s algorithm (Dijkstra 1959).
- *Betweenness *(*shortest path-based*)* centrality* is the proportion of all vertex-pairs shortest paths in a network that pass through a particular vertex (Newman 2008; Freeman 1977). It is used to understand the influence of vertices in the overall flow of information in a network. To compute the betweenness centrality of a vertex $i$, one has to calculate the proportion of shortest paths between two vertices, e.g., $i, j$, that pass through vertex $h$:
  $${\mathrm{C}}_{B}\left(h\right)= \sum_{i\ne h\ne j\in \mathrm{V}}\frac{{\sigma }_{ij}\left(h\right)}{{\sigma }_{ij}} ,$$

where $V$ is a set of vertices, ${\sigma }_{ij}$ is the total number of shortest paths between $i$ and $j$, and ${\sigma }_{ij}\left(h\right)$ is the number of those paths that pass through $h$. For weighted graphs, edges must be greater than zero, and the metric considers the sum of the weights (Fornito et al. 2016). Again, it is necessary to use the distance when using this shortest path-based metric. This formula can also be normalised by putting $\frac{2}{\left(N-1\right)\left(N-1\right)}$ in front of the sum (N being the number of vertices).

### Code example


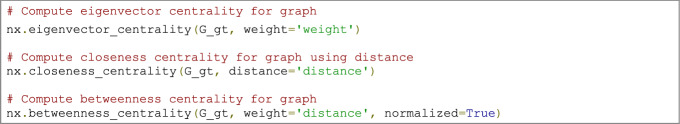


### The minimum spanning tree

The minimum spanning tree is the backbone of a network, i.e., the minimum set of edges necessary to ensure that paths exist between all vertices without forming cycles (Stam et al. 2014; van Dellen et al. 2018). A few main algorithms are used to build the spanning tree, with Kruskal’s algorithm being implemented in *NetworkX* (Kruskal 1956). Briefly, this algorithm ranks the distances between vertices, adds the ones with the smallest distance first, and at each added edge, it checks if cycles are formed or not. The algorithm will not keep an edge that results in the formation of a cycle.

### Code example


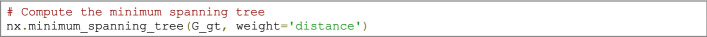


### Modularity

Modularity states how divisible a network is into different modules (or communities). The identification of the modules is performed by the community detection algorithm (Fornito et al. 2016; Meunier et al. 2010; Bullmore and Sporns 2009). Here, we will use the Louvain algorithm (Blondel et al. 2008) as recommended by Fornito et al. (2016). It works in a two-step iterative manner, first looking for communities by optimising modularity locally and then concatenating vertices that belong to the same module (Blondel et al. 2008).

### Code example


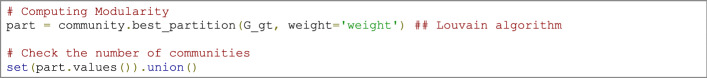


### Topological data analysis

In this section, we will use TDA on our rsfMRI adjacency matrices. TDA can identify different network characteristics by addressing the high-order structure of a network beyond pairwise connections as used in graph theory (Carlsson 2020; Battiston et al. 2020; Kartun-Giles and Bianconi 2019). TDA generally uses topology and geometry methods to study the shape of the data (Carlsson 2009). A core feature of TDA is the ability to provide robust results compared with alternative methods, even if the data are noisy (Blevins and Bassett 2020; Expert et al. 2019). In this context, it is essential to frame the difference between noise and systematic error for applications of TDA properly. Noise is caused by factors that affect the measurement of the variable of interest entirely at random. Systematic errors, however, are not determined exclusively by chance. They are introduced by a factor that systematically influences the variable of interest measurement (e.g. by an inaccuracy involving either the observation or measurement process). In rsfMRI (and other brain-related measures), both types of noise can be present.

One of the benefits of using TDA in network neuroscience is the possibility of finding global properties of a network that are preserved regardless of the way we represent the network (Petri et al. 2014), as we will illustrate below. Those properties are the so-called topological invariants.

**Fig. 3:**
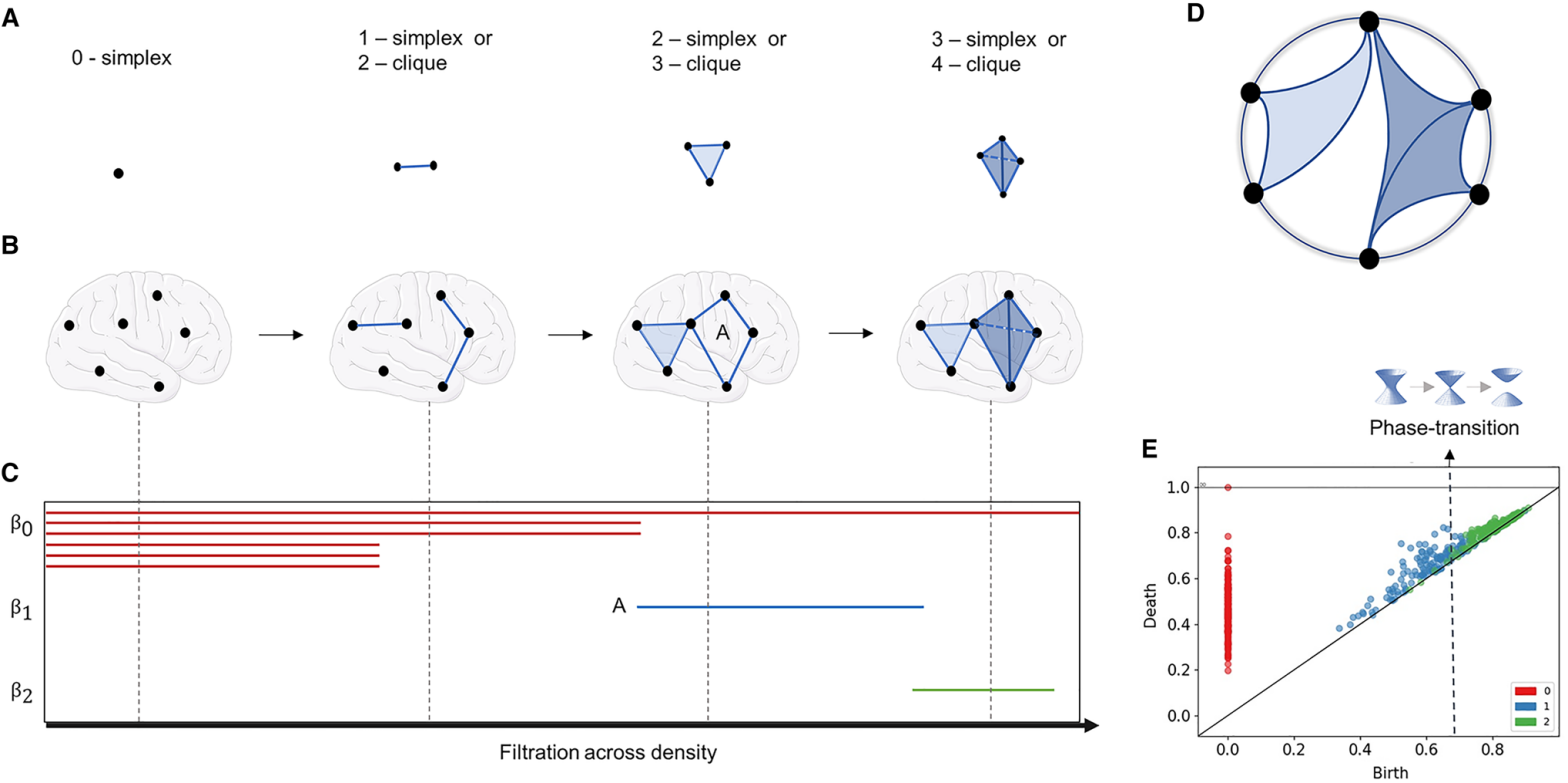
Topological data analysis. **a** Illustration of simplexes. **b** Representation of simplexes/cliques of different order being formed in the brain across the filtration process. **c** Barcode respective to panel *b*, representing the filtration across distances (i.e., the inverse of weights in a correlation matrix). Line A represents cycle A in B. ${\beta }_{0}, {\beta }_{1},$ and ${\beta }_{3}$ indicate the homology groups. (${\beta }_{0}=$ connected components, ${\beta }_{1}$ = one-dimensional holes, ${\beta }_{2}$ = 2-dimensional holes). **d** Circular projection of how the brain would be connected. **e** Persistence diagram (or Birth/Death plot) obtained from real rsfMRI brain data. In this plot, it is also possible to identify a phase transition between ${\beta }_{1}$ and ${\beta }_{2}$

We will cover a few fundamental TDA concepts: filtration, simplicial complexes, Euler characteristic, phase transitions, Betti numbers, curvature, and persistent homology. A summary can be found in Fig. 3.

### The basis: the adjacency matrix and filtration

As indicated in the earlier section on graph theory, there is no consensus on the necessity or level of thresholding performed on rsfMRI-based adjacency matrices. However, TDA overcomes this problem by investigating functional connectivity over all possible thresholds in a network. This process of investigating network properties looking for all possible thresholds instead of choosing a fixed one is called *filtration* (Fig. 3b and Supplementary Material 1). It consists of changing the threshold, e.g., the density *d* of the network, from $0 \le d\le 1$. This yields a nested sequence of networks, in which increasing *d* leads to a more densely connected network. Notice that the notion of filtration is not only used in high order interactions but has also been applied in pair wise, graph-theoretical work (Wang et al. 2018; Gracia-Tabuenca et al. 2020).

### Code example


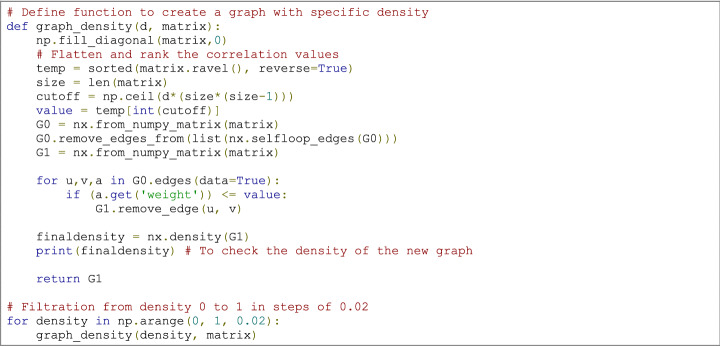


### Simplicial complexes

**Fig. 4:**
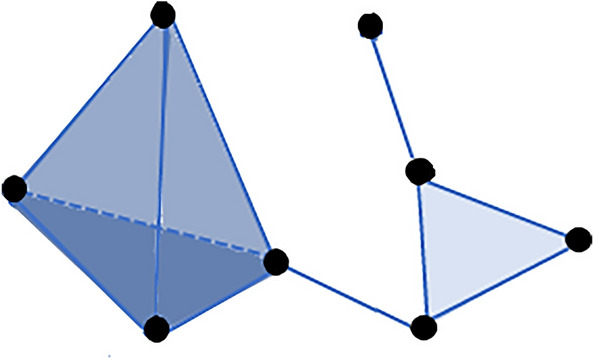
Simplicial complex. An example of a simplicial complex composed of eight vertices (0-simplexes), 11 edges (1-simplexes), five triangles (2-simplexes), one tetrahedron (3-simplexes)

In TDA, we consider that the network as a multidimensional structure called the simplicial complex. Such a network is not only made up of the set of vertices (0-simplex) and edges (1-simplex) but also of triangles (2-simplex), tetrahedrons (3-simplex), and higher *k*-dimensional structures (Fig. 3a). In short, a *k*-simplex is an object in *k-*dimensions and, in our work, is formed by a subset of *k*+*1* vertices of the network (Fig. 4).

We can encode a network into a simplicial complex in several ways (Lambiotte et al. 2019; Edelsbrunner and Harer 2010; Maletić et al. 2008). However, here, we will focus on building a simplicial complex only from the brain network’s cliques, i.e., we will create the so-called clique complex of a brain network. In a network, a *k*-clique is a subset of the network with $k$ all-to-all connected nodes. 0-clique corresponds to the empty set, 1-cliques correspond to nodes, 2-cliques to links, 3-cliques to triangles, and so on. In the clique complex, each *k* + *1* clique is associated with a *k*-simplex. This choice for creating simplexes from cliques has the advantage of using pairwise signal processing to create a simplicial complex from brain networks, such as in Giusti et al. (2015). It is essential to mention that other strategies to build simplicial complexes beyond pairwise signal processing are still under development, such as applications using multivariate information theory together with tools from algebraic topology (Baudot 2019a, b; Barbarossa and Sardellitti 2020; Baudot et al. 2019; Baudot and Bennequin 2015; Rosas et al. 2019; Gatica et al. 2020).

**Fig. 5:**
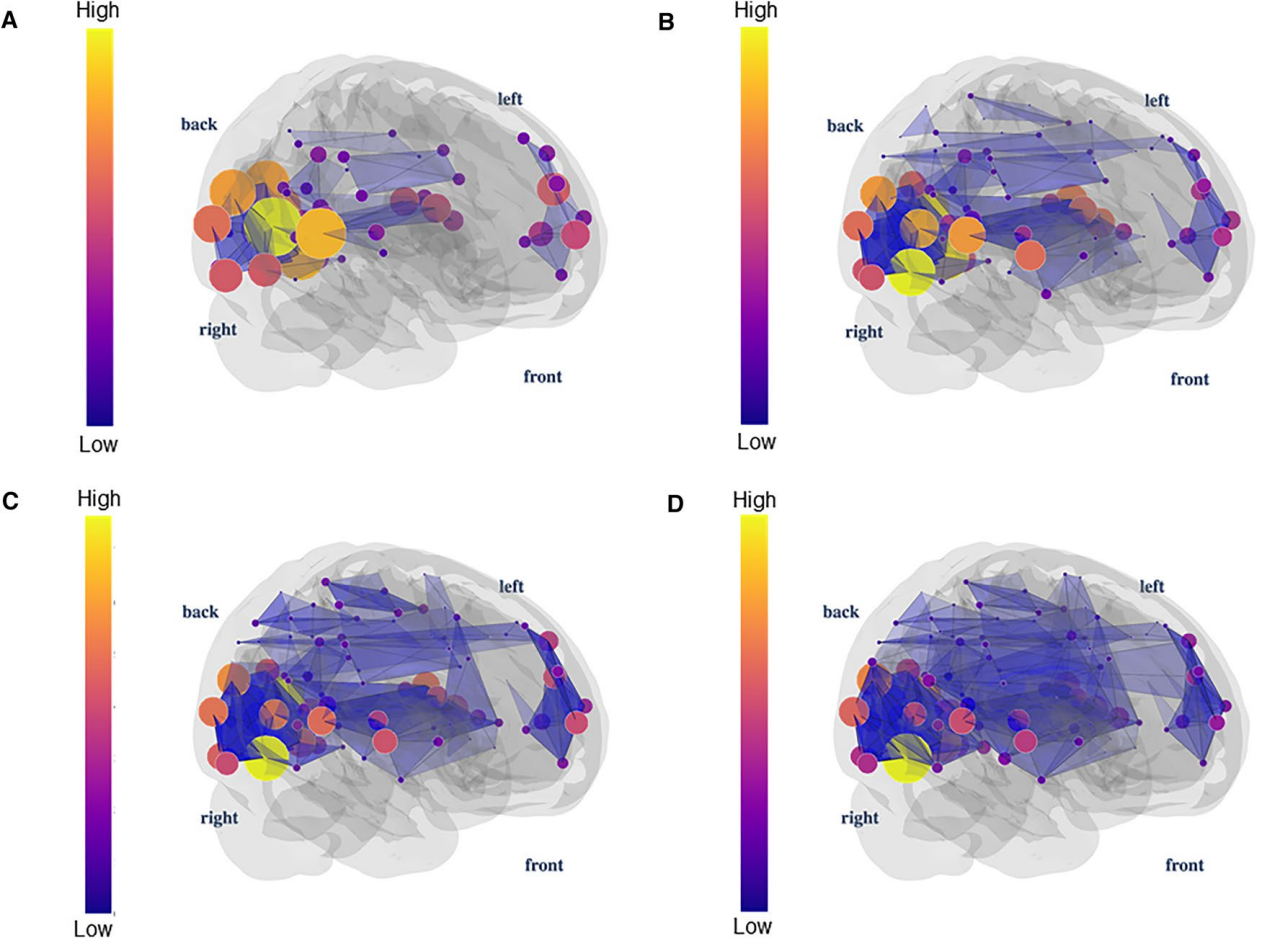
Simplex 3-D visualisation. Here we visualise the rising number of 3-cliques (triangles) in a functional brain network as we increase the edge density *d* (0.01, 0.015, 0.02, and 0.025, from *a* to *d*). For higher densities, we have a more significant number of 3-cliques compared to smaller densities. The vertex colour indicates the clique participation rank

Our Jupyter Notebook provides the code to visualise the clique complex developed in (Santos et al. 2019). To create the 3-D plots, we used mesh algorithms available in *Plotly* (Inc. 2015), together with a mesh surface of the entire brain available in Fan et al. (2016); Bakker et al. (2015). In Fig. 5, we display an example of 3-D visualisation of 3-cliques in the 1000 Functional Connectomes data. When we increase the filtration density *d*, we obtain more connections, and more 3-cliques arise. In Fig. 5, only 3-cliques are shown; however, the same can be done for higher-dimensional cliques like a tetrahedron, *et cetera*. In Supplementary Material 1, we offer filtration in a functional brain network up to 4-cliques. In the Jupyter Notebook, we can also visualise the clique complex at arbitrary sizes, up to computational limits. The computation is not shown here as code blocks due to its size and complexity (see the Jupyter Notebook).

### The Euler characteristic

**Fig. 6:**
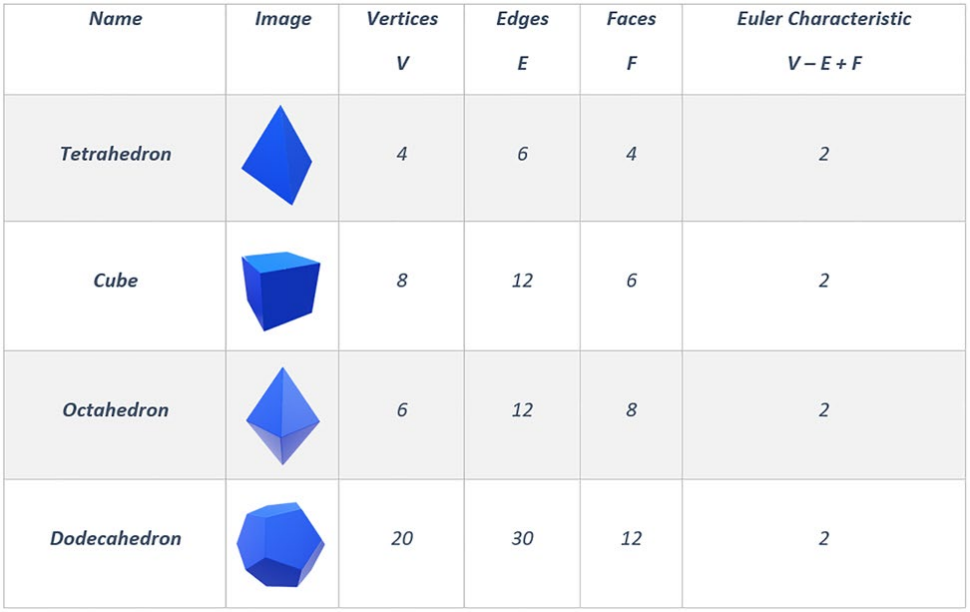
Euler characteristic in convex polyhedra. Note that there are no cavities in their shapes for convex polyhedra, and the Euler characteristic is always equal to two

**Fig. 7:**
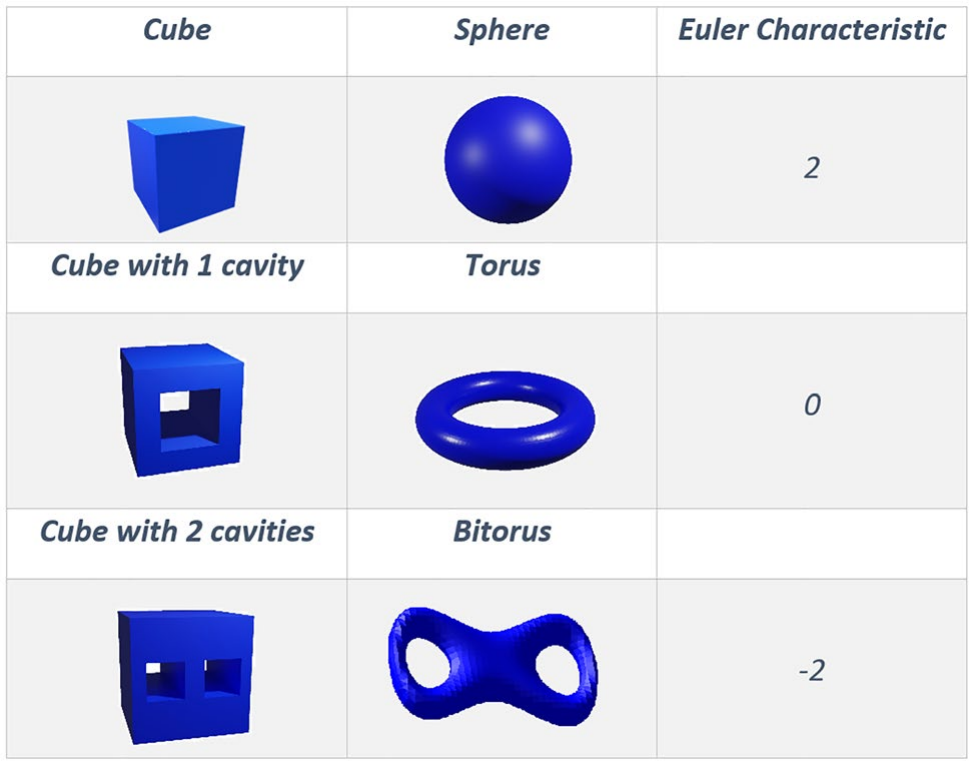
The Euler characteristic in polyhedra with cavities. The Euler characteristic of a cube with a cavity is equal to zero, just as the torus. This value drops to minus two if we have two cavities in the cube, just like a bitorus

The Euler characteristic is one example of topological invariants: the network properties that do not depend on a specific graph representation. We first introduce the Euler characteristic for polyhedra, as illustrated in Fig. 6. Later, we translate this concept to brain networks. In 3-D convex polyhedra (for example, a cube, a tetrahedron, *et cetera*, see Fig 6), the Euler characteristic is defined as the numbers of vertices minus edges plus faces of the considered polyhedra. For convex polyhedra without cavities (holes in its shape), which are isomorphous to the sphere, the Euler characteristic is always two, as you can see in Fig. 6. If we take the cube and make a cavity, the Euler drops to zero as it is in the torus. If we make two cavities in a polyhedral (as in the bitorus), the Euler drops to minus two (Fig. 7). We can understand that the Euler characteristic tells us something about a polyhedron’s topology and its analogous surface. In other words, if we have a surface and we make a discrete representation of it (e.g., a surface triangulation), its Euler characteristic will always be the same, regardless of the way we do it.

We can now generalise the definition of Euler characteristic to simplicial complex in any dimension. Thus, the high dimensional version of the Euler characteristic is expressed by the alternate sum of the numbers ${Cl}_{k }\left(d\right)$ of the *k*-cliques (which are (*k-1*)-simplexes) present in the network’s simplicial complex for a given value of the density threshold *d*.

$$\upchi \left(d\right) = {Cl}_{1}-{Cl}_{2}+\dots {Cl}_{\mathrm{n}}={\sum }_{\mathrm{k}=1}^{\mathrm{n}}{\left(-1\right)}^{\mathrm{k+1}}{Cl}_{k }\left(d\right).$$

### Code example


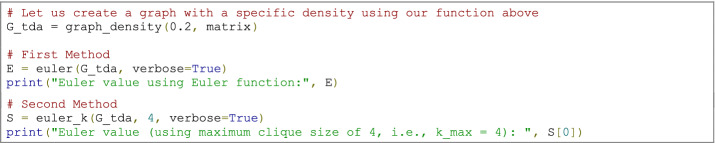


Note that the clique algorithm, the primary function used in our code (euler—Supplementary Material 2), is an NP-complete problem, which is computationally expensive for large and/or dense networks, regardless of how you implement it (Pardalos and Xue 1994). An alternative is to fix an upper bound for the cliques’ size (Pardalos and Xue 1994; Gillis 2018). Therefore, the second function (*euler_k*—Supplementary Material 2) allows the user to constrain the maximum size of the cliques we are looking for. This means that we are fixing the dimension *k* of our simplicial complex and ignoring simplexes of dimension greater than *k*.

### Topological phase transitions

Phase transitions can provide insight into the proprieties of a ‘material’. For example, water is known for becoming steam at 100 °C. Similarly, by using TDA when comparing a patient and healthy population, one could identify these populations’ properties by studying each group’s topological phase transition profile. This strategy has already been applied for investigating group differences between controls and glioma brain networks (Santos et al. 2019) and typically developing children and children with attention-deficit/hyperactivity disorder (Gracia-Tabuenca et al. 2020). In other fields, topological phase transitions were also investigated in the *S. cerevisiae* and *C. elegans protein interaction networks,* reionisation processes, and evolving coauthorship networks (Amorim et al. 2019; Giri and Mellema 2021; Lee et al. 2021).

To investigate topological phase transitions in brain networks, we first need to visualise the *Euler entropy* (Fig 3 in Santos et al. 2017):

${S}_{\upchi }$= ln|$\upchi$ |.when $\upchi$ = 0 for a given value of the density of the network, the Euler entropy is singular, ${S}_{\upchi } \to \infty$. Under specific hypotheses, a topological phase transition in a complex network occurs when the Euler characteristic is null (Santos et al. 2019). This statement finds support in the behaviour of ${S}_{\upchi }$ at the thermodynamic phase transitions across various physical systems (Santos et al. 2017). In network theory, the Giant component transition is associated with network changes, from smaller connected clusters to the emergence of Giant ones (Erdős 1959). Theoretically, topological phase transitions are related to the extension of the Giant component transition for simplicial complexes (Linial and Peled 2016). Based on numerical simulations, it was also conjectured that the longest cycle is born in the phase transition vicinity (Bobrowski and Skraba 2020; Speidel et al. 2018). Phase transitions can also be visualised in Birth/Death plots (Fig. 3e) which will be discussed later in the Persistent Homology section.

### Betti numbers

Another set of topological invariants are the Betti numbers ($\beta$). Given that a simplicial complex is a high-dimensional structure, ${\beta }_{k}$ counts the number of *k*-dimensional holes in the simplicial complex. These are topological invariants that correspond, for each $k\ge$ 0, to the number of linearly independent *k*-dimensional holes in the simplicial complex (Zomorodian 2005).

**Fig. 8:**
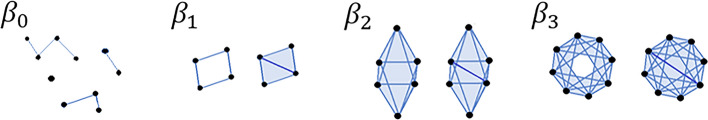
Betti numbers and examples of each *k*-dimensional hole. ${\beta }_{0}$ is the number of connected components or zero-dimensional holes. ${\beta }_{1}$ is the number of one-dimensional holes (loops). ${\beta }_{2}$ is the number of two-dimensional holes (voids). ${\beta }_{3}$ is the number of 3-D holes. For ${\beta }_{1}$, ${\beta }_{2}$, ${\beta }_{3}$ only the left figure of each pair represents the k-dimensional hole. In the right figure, a connection is added, and so the k-hole is lost: the right figure of each pair no longer represent a $\beta$ hole. For ${\beta }_{0}$ the number of connected components is the number of separate clusters we have in the figure; therefore, we should consider the figure as a whole (in the case represented here, we have four connected components)

In Fig. 8, we show the representation of the *k*-dimensional holes. We give one example for each dimension. In a simplicial complex, there can be many of these *k*-holes and counting them provide the Betti number $\beta$, e.g., if ${\beta }_{2}$ is equal to five, there are 5 two-dimensional holes.

### Code example


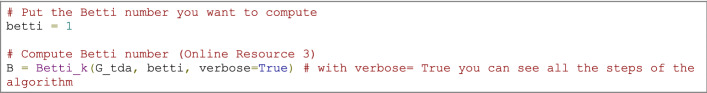


Notice that for higher k or dense simplicial complexes, the calculation of the $\beta$ becomes computationally expensive.

There are ways to estimate the $\beta$ of a simplicial complex without calculating it directly. It is known that the $\beta$ relate to Euler characteristics and phase transitions. The Euler characteristics of a simplicial complex can also be computed using the $\beta$ via the following formula (Edelsbrunner and Harer 2010):

$$\upchi ={\beta }_{0}-{\beta }_{1}+{\beta }_{2}-\dots {{\left(-1\right)}^{{\mathrm{k}}_{\mathrm{max}}}\beta }_{{\mathrm{k}}_{\mathrm{max}}}={\sum }_{\mathrm{k}=0}^{{\mathrm{k}}_{\mathrm{max}}}{\left(-1\right)}^{\mathrm{k}}{\beta }_{k },$$

where $k\_\mathrm{max}$ is the maximum dimension that we are computing the cycles.

Furthermore, topological phase transitions are also defined as the $\beta$ of a simplicial complex (Bobrowski and Kahle 2018). We know that ${\upbeta }_{0}$ counts the number of the connected components of a simplicial complex. Suppose we compute the Betti curves as a function of probability in stochastic models. In that case, each Betti curve passes through two distinct phases in a narrow interval: one when it first emerges and the other when it vanishes (Linial and Peled 2016). That means that, under similar assumptions as in theoretical models, if the $\beta$ distribution is unimodal, increasing the density of edges of a brain network will lead to the appearance of $\beta$ of a higher order. In contrast, smaller Betti numbers will disappear at the vicinity of a topological phase transition.

**Fig. 9:**
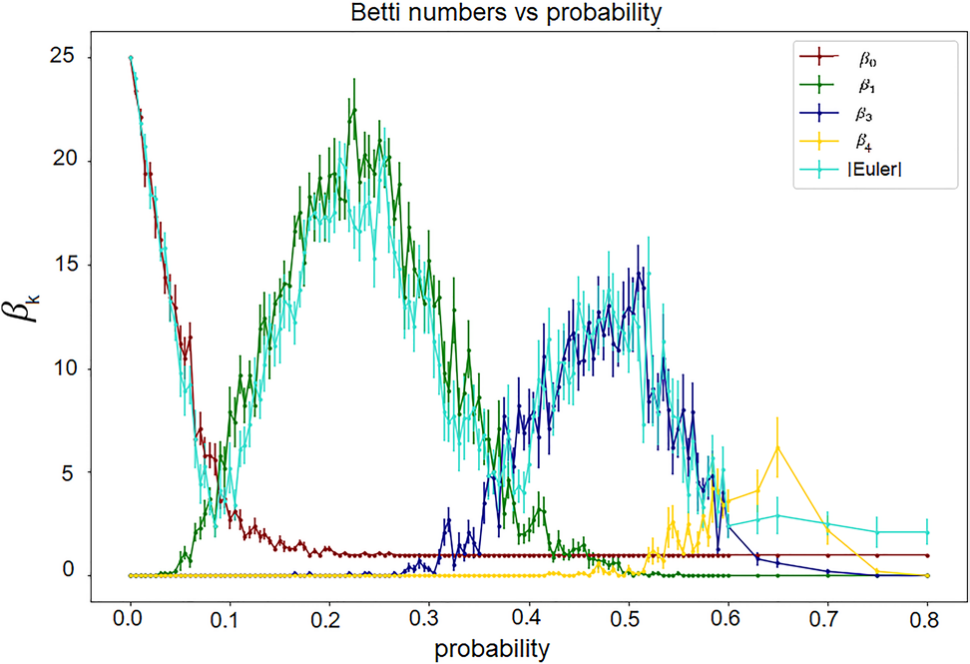
Betti number and Euler characteristic approximation. We create a random network for each probability of connection between vertices, and we compute the $\beta$ and the absolute value of the Euler characteristic. We repeated the experiment 10 times and calculated the mean curves with errors. This plot only shows the mean (with errors) of the ten experiments for the Euler and Betti curves. We notice that the absolute value of the Euler characteristic is a good approximation of $\beta$

In Fig. 9, we illustrate this property on simplicial complexes obtained from random networks. As the probability increases and so the density of the network is higher, we find a sequence of dominant ${\beta }_{k}$ starting from *k* = 0, that change (i.e., *k* is incremented by one unity) every time a topological phase transition occurs. While the singularities of the Euler entropy ${S}_{\upchi }$ determine the transitions’ location, the crossover of the $\beta$ characterises which kind of multidimensional hole prevails in each topological phase of the filtration.

### Curvature

Curvature is a TDA metric that can link the global network properties described above to local features (Weber et al. 2017; Farooq et al. 2019; Santos et al. 2019). When working with brain network data, this will allow us to compute topological invariants for the whole-brain set of vertices and understand the contribution of specific individual nodal, or subnetwork, geometric proprieties to global properties of the brain network.

Several approaches to defining a curvature for networks are available (Najman et al. 2017; Weber et al. 2017), including some already used in neuroscientific investigations (Santos et al. 2019). We will illustrate the curvature approach linked to topological phase transitions, previously introduced for complex systems in (Farooq et al. 2019; Najman et al. 2017; Wu et al. 2015).

To compute the curvature (Supplementary Material 4), filtration is used to calculate the clique participation rank (i.e., the number of $k$-cliques in which a vertex $i$ participates for density *d*) (Sizemore et al. 2018), which we denote here by ${Cl}_{ik }\left(d\right)$. The curvature of the vertex based on the participation rank is then defined as follows:

$$\kappa \_i = \sum_{k=1}^{{k}_{max}}{\left(-1\right)}^{k+1}\frac{{Cl}_{ik }\left(d\right)}{k} ,$$

where ${Cl}_{ik}$ = 1 since each vertex $i$ participates in a single 1-clique (the vertex itself), and ${k}_{max}$ the maximum number of vertices that are all-to-all connected in the network.

To link this nodal curvature to the network’s global properties, we use the Gauss-Bonnet theorem for networks, through which one can connect a local curvature of a network and its Euler characteristic. Conversely, by summing up all the curvatures of the network across different thresholds, one can reach the alternate sum of the numbers $C{l}_{k}$ of *k*-cliques (a subgraph with *k* all-to-all connected vertices) present in the simplicial complex of the network for a given density threshold d ∈ [0, 1], according to the following equation:

$$\chi \left(d\right) = \sum_{k=1}^{N}{\left(-1\right)}^{k+1}{Cl}_{k }\left(d\right).$$

By doing so, we also write the Euler characteristics as a sum of the curvature of all network vertices, i.e.,

$$\upchi \left(d\right) = {\sum }_{\mathrm{i}=1}^{\mathrm{N}}{k}_{i}\left(d\right).$$

**Fig. 10:**
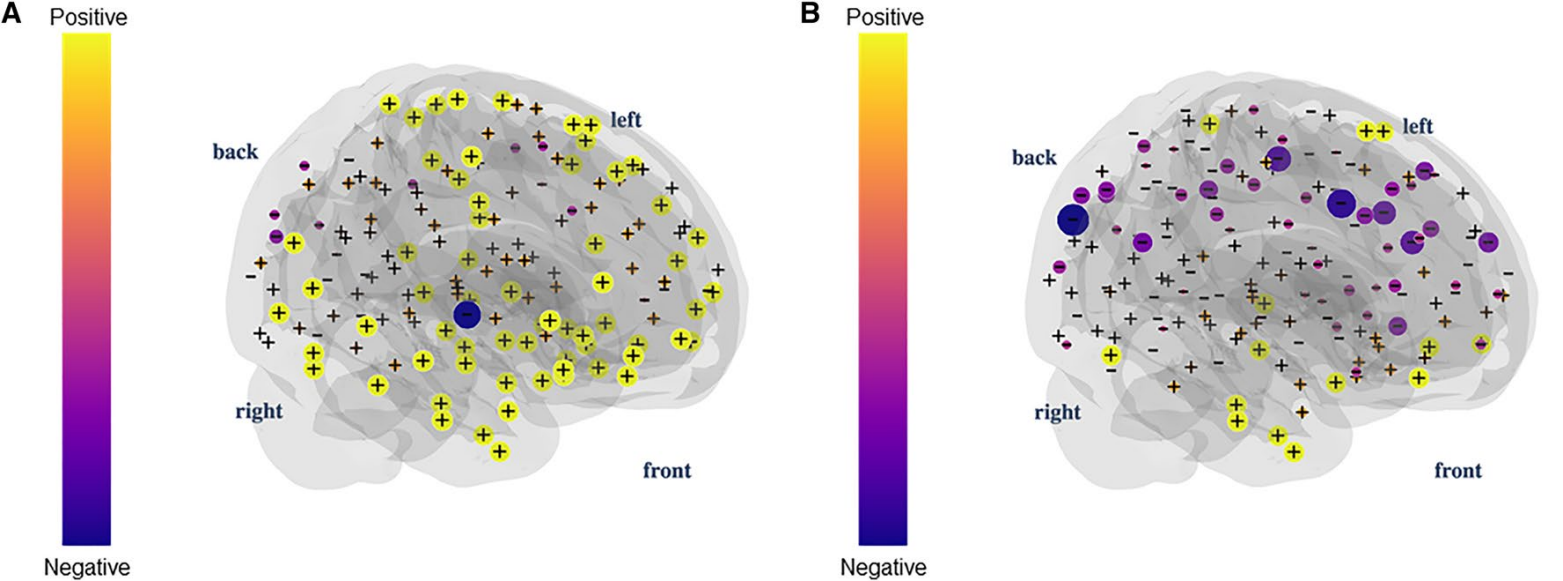
Curvature 3-D plot. Distribution of curvatures in a functional brain network for densities 0.01 (**a**) and 0.03 (**b**) after the first topological phase transition. The sum of curvature over all vertices is equal to the Euler characteristic

We illustrate the curvature distribution for a functional brain network for densities before and after the transition in Fig. 10.

### Persistent homology

Homology is a topology branch that investigates objects’ shapes by studying their holes (or cycles). Persistent homology tracks the emergence of cycles across the evolving simplicial complexes during filtration, allowing us to recognise whether there were homology classes that “persisted” for many filtrations (time here meaning the threshold gap between the birth and death of a cycle) (Curto 2017; Giusti et al. 2016). Importantly, to compute persistent homology, we need to work with a distance matrix, the first step in the code below. We can then calculate the simplicial complex’s persistence and plot it as a barcode or a persistence diagram (Fig. 3c and e). Here we used the *Gudhi* package for the implementation of those steps (Maria et al. 2014). The topological phase transitions in complex networks (Amorim et al. 2019; Santos et al. 2019) can also be identified between the changes in the dimensionality of the birth/death graphs mentioned above (Fig. 3e).

### Code example


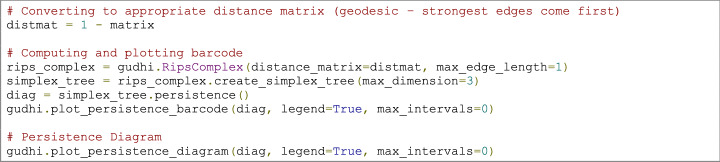


## Discussion

This tutorial has explained some of the main metrics related to two network neuroscience branches—graph theory and TDA—providing short theoretical backgrounds and code examples accompanied by a publicly available Jupyter Notebook. We innovate by combining hands-on explanations with ready-to-use codes of these subfields and visualisations of simplicial complexes in the brain, hopefully lowering the high threshold necessary for neuroscientists to get acquainted with these new analysis methods, particularly for these new methods rsfMRI data. Here, we also innovate by providing realistic visualisation of higher-order simplices in brain networks.

Our main goal was to provide a step-by-step computational tutorial to use graph theory and TDA on brain imaging data, particularly rsfMRI, with in-depth explanations behind each metric. The core idea of applying these analysis frameworks to brain data is that both frameworks can quantitatively combine two evidently essential characteristics of the brain: the brain not only works both at a local level in specialised brain regions but also contains apparent global properties that are of importance for its functioning, which are usually investigated in isolation. As a potentially powerful fusion between localizationism and holism, graph theory and TDA concepts have already been applied in brain research. Starting with graph theory, all the metrics mentioned above have been used in the investigation of brain networks in both normal or pathological states (Eijlers et al. 2017; Garcia-Garcia et al. 2015; Wang et al. 2017; Wink 2019; Breedt et al. 2021; DeSalvo et al. 2020; Liu et al. 2012; dos Santos Siqueira et al. 2014; Yu et al. 2012; Davis et al. 2013; Suo et al. 2015). As one can identify by reading these articles, researchers often use different graph-theoretical metrics in the same study, which helps them look for alterations that might explain group differences in specific contexts (gender, age, pathology, development). This brief review and commentary (Eijlers et al. 2019) summarise some applications. Now, moving on to the newer framework of TDA in neuroscience, fewer studies have been published using rsfMRI data. Santos et al. (2019) applied the concepts of the Euler characteristic, topological phase transitions and curvature in human brain data, to show that these transitions can be found in brain data, helping pave the way for TDA in brain data applications.

Moreover, alterations in whole-brain connectomes were identified in attention-deficit/hyperactivity disorder subjects using Betti numbers and persistent homology, complementing connectomics-related methods that aim to identify the markers of this disorder (Gracia-Tabuenca et al. 2020). A similar approach was used in an Alzheimer’s disease dataset by Kuang et al. (2019). More considerations on how TDA can be used in brain imaging big data and resting-state functional connectivity analyses can be found in Phinyomark et al. (2017); Petri et al. (2014); Anderson et al. (2018); Saggar et al. (2018); Salch et al. (2021); Songdechakraiwut and Chung (2020).

Notably, limitations and other relevant points should be kept in mind when working with these metrics. Firstly, it is common in network neuroscience to use null models for comparison with real data. The idea is to show that the results are different from what one would obtain by chance (or randomly). The generation and comparison with null models must be performed differently for graph theory and TDA, and it is crucial to define what propriety should be kept constant (e.g., the density of the network or degree distribution). For instance, in Viger and Latapy (2005), if one wants to generate null models with a prescribed degree sequence. In this context, simplicial complexes built from Erdo-Renyi networks illustrated in Fig. 9 are the simplest (and by no means realistic) null models we can generate.

Nevertheless, the computation and discussion of null models are beyond this tutorial’s scope and would be an article in itself. A more in-depth discussion of null models in graph theory can be found in Fornito et al. (2016). Please see Sect. 4 of Battiston et al. (2020) and Blevins and Bassett (2020) for null models in simplicial complexes.

Moreover, it is crucial to appreciate limitations in interpretation when using these metrics in connectivity-based data. Since rsfMRI data is often calculated as a temporal correlation between time series using Pearson’s correlation coefficient, a bias on the number of triangles can emerge. For example, suppose areas A and B and areas C and B are communicating and thus correlated. In that case, a correlation will be present between A and C, even if there would be no actual communication between these vertices (Zalesky et al. 2012). This can affect graph-theoretical metrics such as the clustering coefficient, with networks based on this statistical method being automatically more clustered than random models, and TDA metrics, where the impact depends on how high-order interactions are defined. The proper way to determine and infer high-order interactions in the brain is an ongoing challenge in network neuroscience. Here we simplified our approach using the cliques of a network to define our simplicial complex. For those interested in a more in-depth discussion on the topic, we recommend Sects. 1 and 3 of chapters 7 and 10, respectively, in Fornito et al. (2016).

The use of weighted matrices can also come with caveats. As mentioned above, various metrics use the sum of weights to compute final nodal values. From that, multiple edges with low weights might have a final sum equal to a few edges with higher weights. How to deal with this limitation and distinguish between these cases is still under discussion. A possible solution was proposed by Opsahl et al. (2010), in which the addition of a tunable parameter in the computation of centralities can allow the researcher to include the number of edges in the total sum, not only the sum of the weights.

Concerning TDA, it is essential to think about limitations in its use due to computational power. The computation of cliques falls in the clique-problem, an NP (nonpolynomial time) problem, thus listing cliques may require exponential time as the size of the cliques or networks grows (Gillis 2018; Pardalos and Xue 1994). For example, if the matrix to be analysed has 60 vertices with a maximum clique size of 23, this will correspond to $\sum \left(\genfrac{}{}{0pt}{}{60}{k}\right)$ for $k \in \left\{0,\dots , 23\right\}$ cliques, resulting in an enormous amount of time to compute all cliques. What we can do for practical applications is to limit the clique size that can be reached by the algorithm, which determines the dimension of the simplicial complex in which the brain network is represented. This arbitrary constraint implies a theoretical simplification, limiting the space or the dimensionality in which we would analyse brain data. Another issue is that, to finish TDA computations in a realistic timeframe, the researcher might need to establish a maximal threshold/density for convergence even after reducing the maximal clique size. Even though TDA approaches lead to substantial improvements in network science; apart from applications using the Mapper algorithm (Saggar et al. 2018), the limitations mentioned above contribute to losing information on the data’s shape (Stolz 2014).

Furthermore, given the early stage of TDA approaches in clinical network neuroscience, it is relevant to recognise that the neurobiological meaning of the metrics mentioned here is still limited. Further studies contrasting different neuroscientific techniques with TDA must be done to improve the understanding, in the neurobiological level, on what a topological metrics represent and how they correlate with brain functioning. However, it is already possible to use these metrics to differentiate groups (Santos et al. 2019; Gracia-Tabuenca et al. 2020), and plausible to assume that the interpretation of some classical metrics could be extrapolated to higher orders interactions. For example, the concept of the centralities using pairwise interactions is used to understand node importance and hubs, the same, in theory, could be applied to the relationships between 3 or more vertices by extending the definition of centrality from networks to simplicial complexes, as done in Hernández Serrano and Sánchez Gómez (2020); Estrada and Ross (2018).

Last, we would like to briefly mention more general problems in network neuroscience and brain imaging. Before applying graph theoretical or topological data analysis, one should be aware of frequent arbitrary decisions such as defining thresholds, using binary or weighted matrices, and controlling for density. Besides, one should think about the differences that arise from using particular atlases and parcellations and their influence on the findings (Wang et al. 2009; Douw et al. 2019; Fornito et al. 2016; Gracia-Tabuenca et al. 2020; Wu et al. 2019; Eickhoff et al. 2018; Bullmore and Sporns 2009). All these factors can impact how credible and reproducible the field of network neuroscience will be, inevitably influencing how appealing the metrics’ use might be to clinical practice (Douw et al. 2019).

## Conclusion

Network neuroscience is pivotal in the understanding of brain organisation and function. Graph theory has been the most utilised framework so far, but as the field of network neuroscience expands, newer methods such as TDA are starting to take part in the investigation. To further improve the field, especially in clinical network neuroscience, it is imperative to make the computation of the developed metrics accessible, easy to comprehend, visualise, and efficient. Moreover, researchers must be aware of the crucial decisions one must make when executing data analysis and how these can affect studies’ results and reproducibility. We hope to have facilitated the comprehension of some aspects of network and topological neuroscience, the computation and visualisation of some of its metrics. As a final reminder, we would again suggest the reader to explore our table of resources and the Jupyter Notebook developed by our team.

## Supplementary Information

Below is the link to the electronic supplementary material.

Supplementary file1 (MP4 11625 KB) Supplementary Material 1. Video. 3-D filtration of networks with 1, 2, 3, and 4-cliques

Supplementary file2 (DOCX 47 KB) Supplementary Material 2. Code block. Computation of the Euler characteristic. Supplementary Material 3. Code block. Computation of the Betti numbers. Supplementary Material 4. Code block. Computation of Curvature

## Data Availability

Not applicable.

## Footnotes

1 Notice that the notion of metric in mathematics defines distance between two points in a set (Do Carmo 2016), which is distinct from what we are using in this work. We denote as metric any quantity that can be computed, *i.e.*, “measured”, in a brain network or simplicial complex.

## Code availability

https://github.com/multinetlab-amsterdam/network_TDA_tutorial; https://zenodo.org/record/4483651.
