# Supplementary material for: A hands-on tutorial on network and topological neuroscience
Source: Brain Struct Funct. 2022 Feb 10;227(3):741–62. doi: 10.1007/s00429-021-02435-0 (PMC8930803; doi:10.1007/s00429-021-02435-0)
Supplement: Supplementary file 2 — Supplementary file2 (DOCX 47 KB) Supplementary Material 2. Code block. Computation of the Euler characteristic. Supplementary Material 3. Code block. Computation of the Betti numbers. Supplementary Material 4. Code block. Computation of Curvature [file 429_2021_2435_MOESM2_ESM.docx]

**Online Resource 2. Code block. Computation of the Euler characteristic.**

# Function to compute the Euler Characteristic

def euler(G, verbose=False):

"""Function to compute the Euler characteristic of the clique complex of

a network

Parameters

----------

G: networkx graph

Returns

-------

ec: int

value of the Euler characteristic of the clique complex of the network

Notes

-----

If verbose = True, it will print all the steps of the calculation, so

that one can check whether the code is working well, or different stages

of the computation. Otherwise, if verbose=False, only the output will be

returned.

"""

def DIAGNOSTIC(*params):

if verbose:

print(*params)

DIAGNOSTIC("Nodes in G: ", G.nodes())

DIAGNOSTIC("Edges in G: ", G.edges())

DIAGNOSTIC("Number of nodes: {}, edges: {}".format(G.number_of_nodes(),

G.number_of_edges()))

# Compute maximal cliques

C = nx.find_cliques(G)

# Create list C with all the cliques

# Sort each clique, convert it from list to tuple

C = [tuple(sorted(c)) for c in C]

DIAGNOSTIC("List with all maximal simplexes/cliques C:",C)

DIAGNOSTIC("Number of maximal cliques: %i"%(len(C)))

# Enumerate all simplices/cliques

S = []

n = max(len(c) for c in C)

for k in range(0, n) :

# Get all (k+1)-cliques, i.e. k-simplices, from max cliques mc

Sk = sorted(set(c for mc in C for c in itertools.combinations(mc,

k+1)))

DIAGNOSTIC("list of %i-simplex S%i:"%(k,k), Sk)

# Check that each simplex is in increasing order

assert(all((list(s) == sorted(s)) for s in Sk))

# Assign an ID to each simplex, in lexicographic order

S.append(dict(zip(Sk, range(0, len(Sk))))) # zip(Sk,range()) is an

object (composed by tuples) where each element of Sk is associated to

a number. Then from the zip object create the dictionary where the

key is the Sk element and the value the number.

for (k, Sk) in enumerate(S):

DIAGNOSTIC("Number of {}-simplices: {}".format(k, len(Sk)))

DIAGNOSTIC("S dictionary", S)

# The cliques are redundant now

del C

# Euler characteristic

ec = sum(((-1)**k * len(S[k])) for k in range(0, len(S))) # Alternate sum

of all the simplexes/cliques of different dimensions. len(S[k])

is how many k-simplex we have. len(S) is the maximum dimension k we can

find (i.e. the dimension of the simplicial complex)

DIAGNOSTIC("Euler characteristic:", ec)

return ec

# Function to compute the Euler characteristic of the clique complex of the network with the constraint that we look for cliques up to max dimension k

def euler_k(G, kmax, verbose=False):

"""Function to compute the Euler characteristic of a network with the

constraint that we look for cliques up to max dimension k

Parameters

----------

G: networkx graph

kmax: int

clique maximum dimension

Returns

-------

Possibilities:

S: list

0:Euler characteristic, 1:total cliques, 2:maximum dimension of clique,

3:number of clique_0, 4:Clique_1, 5:Clique_2, 6:Clique_3, and so on …

Notes

-----

If verbose = True, it will print all the steps of the calculation, so

that one can check whether the code is working well. Otherwise, if

verbose=False, only the output will be returned.

"""

# Prepare maximal cliques

Nodes = len(G)

Cliques = nx.find_cliques(G)

# This function computes the max number for cliques that exists for a

given size

def max_cliques(N, k):

mclq = 0

for i in range(0, k):

mclq += scipy.special.binom(N, k)

return int(mclq)

Limit = max_cliques(Nodes,kmax) #maximum number of cliques that you

want to find

if verbose == True:

print("Limit:", Limit)

Cl = []

while True:

try:

for i in range(0,Limit):

clq=next(Cliques)

if len(clq)<= kmax:

Cl.append(clq)

except StopIteration:

break

# Sort each clique, make sure it is a tuple

C = [tuple(sorted(c)) for c in Cl]

if verbose== True:

print("C:",Cl)

S = [] # Will contain the number of clique of each order k

for k in range(0, max(len(s) for s in C)):

# Get all (k+1)-cliques, i.e. k-simplices, from max cliques mc

Sk = set(c for mc in C for c in itertools.combinations(mc, k+1))

S.append(len(Sk))

tau = sum(S) # Tau gives the total number of cliques

kmax = len(S) # Kmax is the maximum clique size one can find

if verbose == True:

print("total # of cliques:", tau)

print("maximum clique size we can find:", kmax)

Ec = 0 # Ec is the Euler characteristic

Ec = sum(((-1)**i * S[i]) for i in range(0, len(S)))

if verbose== True:

print("The Euler characteristic EC is:", Ec)

  S.insert(0, kmax)

S.insert(0, tau)

S.insert(0, Ec)

if verbose== True:

print("S: Euler, total cliques, maximum dimension of clique, number

of clique_0,Clique_1,Clique_2, Clique_3")

print("S:", S)

# We want to include new elements after kmax with zero, to say that there

are no simplices with this size – We fixed the elements to 30, this is

flexible

for i in range(kmax, 30):

S.insert(kmax+3, 0)

return S # The output will be EC, tau, kmax, clique_0,Clique_1,Clique_2,

Clique_3, and so on…

**Online Resource 3. Code block. Computation of the Betti numbers.**

# Function to compute the desired Betti number of the clique complex of the network

def Betti_k(G, K_input, verbose=False):

"""Function to compute the desired Betti number of a network

Parameters

----------

G: networkx graph

K_input: int

0 if you want to compute Betti-0, 1 if you want to compute Betti-

1, 2 for Betti-2 etc. Notice that, the higher the K_input, more

complex/time consuming is the computation.

Returns

-------

B: int

Betti number

Notes

-----

If verbose = True, it will print all the steps of the calculation, so

that one can check whether the code is working well. Otherwise, if

verbose=False, only the output will be returned.

"""

def DIAGNOSTIC(*params):

if verbose:

print(*params)

DIAGNOSTIC("Nodes in G: ", G.nodes())

DIAGNOSTIC("Edges in G: ", G.edges())

DIAGNOSTIC("Number of nodes: {}, edges: {}".format(G.number_of_nodes(),

G.number_of_edges()))

# Compute maximal cliques

C = nx.find_cliques(G)

# Create list C with all the cliques

# Sort each clique, convert it from list to tuple

C = [tuple(sorted(c)) for c in C]

DIAGNOSTIC("List with all maximal simplex C:", C)

DIAGNOSTIC("Number of maximal cliques: %i"%(len(C)))

# Enumerate all simplices

S = [] # Will be a list of dictionaries

# Setting the range

if K_input == 0:

ini = 0

fin = 2

else:

ini = K_input-1

fin = K_input+2

DIAGNOSTIC("I start the loop where I create the required Sk to then

compute Betti. Sk is a list with the k-simplex")

for k in range(ini, fin): # k has 2 values for betti_0 and 3 values for

betti1_2_3

Sk = sorted(set(c for mc in C for c in itertools.combinations(mc,

k+1)))

DIAGNOSTIC("list of %i-simplex S%i:"%(k, k), Sk)

# Check that each simplex is in increasing order

assert(all((list(s) == sorted(s)) for s in Sk))

# Assign an ID to each simplex, in order

S.append(dict(zip(Sk, range(0, len(Sk))))) # zip(Sk,range()) is an

object (composed by tuples) where each element of Sk is associated to

a number. Then from the zip object create the dictionary where the

key is the Sk element and the value the number.

DIAGNOSTIC("Number of %i-simplices: "%(k), len(Sk))

DIAGNOSTIC("S dictionary", S)

#The cliques are redundant now

del C

# Construct the boundary operator/matrix

# Boundary Matrix

D = [None, None] # List with the two different k-boundary operators

if K_input == 0:

# D[0] is the zero matrix

D[0] = (np.zeros((1, G.number_of_nodes()))

for k in range(1, len(S)):

# Set the index of D[] and the number of nodes in each group for the

combinatory part

if K_input == 0:

index = k

b = k

else:

index = k-1

b = k+(K_input-1)

# Create a matrix of size (len(S[k-1]), len(S[k])

D[index] = np.zeros( (len(S[k-1]), len(S[k])) )

for (ks, j) in S[k].items() :

a = sorted(itertools.combinations(ks, b))

# Indices of all (k-1)-subsimplices s of the k-simplex ks

I = [S[k-1][s] for s in sorted(itertools.combinations(ks, b))]

for i in range(0,len(I)):

D[index][I[i]][j] = (-1)**(i)

if D[index].shape[1] == 0:

DIAGNOSTIC("I can't create matrix D because the simplicial

complex does not have the needed k-simplex")

DIAGNOSTIC("Boundary matrix:")

DIAGNOSTIC("D",D[index])

DIAGNOSTIC("D_{} has shape {}".format(K_input, D[0].shape))

DIAGNOSTIC("D_{} has shape {}".format(K_input+1, D[1].shape))

# The simplices are redundant now

del S

# Compute rank and dim(ker) of the boundary operators

# dim(Im)= Rank and dim(ker) = V-rank

rank = [0 if d.shape[1] == 0 else np.linalg.matrix_rank(d) for d in D]

ker = [(d.shape[1] - rank[n]) for (n, d) in enumerate(D)]

#The boundary operators are redundant now

del D

DIAGNOSTIC("ker:", ker)

DIAGNOSTIC("rank:", rank)

# Compute the Betti number

# Betti number

B = ker[0]-rank[1]

DIAGNOSTIC("Betti= ker[0]-rank[1]")

DIAGNOSTIC("End of computation\nBetti %i is:"%K_input, B)

return B

**Online Resource 4. Code block. Computation of Curvature.**

def Kmaxcliques(G, kmax=kmax):

"""

Parameters

----------

G: networkx graph

kmax: int

number of dimensions

Returns

-------

C: list with all cliques of the graph G with size up to kmax

"""

Nodes = len(G)

Cliques = nx.find_cliques(G)

Limit = max_cliques(Nodes, kmax)

Cl= []

while True:

try:

for i in range(0, Limit):

clq = next(Cliques)

if len(clq) <= kmax: # IF YOU DON'T WANNA USE KMAX JUST C

COMMENT THIS STEP TO MAKE IT QUICKER

Cl.append(clq)

except StopIteration:

break

# Sort each clique, make sure it's a tuple

C = [list(sorted(c)) for c in Cl]

return C

def Kmax_all_cliques(G, kmax=kmax):

"""Enumerate all cliques to a max size

"""

C = Kmaxcliques(G)

Sk = set()

for k in range(0, max(len(s) for s in C)) :

#Get all (k+1)-cliques, i.e. k-simplices, from all max cliques mc in C

# Notice that we are usning set(c) so that we count each clique only once

[Sk.add(c) for mc in C for c in itertools.combinations(mc, k+1)]

# Check that each simplex is in increasing order

#Appending the number of cliques of size k+1

Cliques = [list(i) for i in Sk]

return Cliques

# Compute nodal curvature based on density

def Curv_density(d, matrix, verbose=False):

"""Compute nodal curvature based on density

Parameters

---------

d: float

density value

matrix: numpy matrix

connectivity matrix

Returns

-------

curv: numpy array

array with curvature values

"""

def DIAGNOSTIC(*params) :

if verbose : print(*params)

DIAGNOSTIC("This function run over all nodes and computes the curvature

of the nodes in the graph")

# This is the initial Graph

G = graph_density(d, matrix) # Filtration function

temp = Kmax_all_cliques(G)

# This lista is a vector V where each v_i is the number of cliques of

size i

lista = []

# We suppose that the size of the cliques are smaller than 20, so we

create an empty list of size 20 for the lista

for i in G.nodes():

lista.append([0] * 50) # creating a list of lists for each node – all

empty for the scores for each size for each node

DIAGNOSTIC("These are all cliques of the Network:")

DIAGNOSTIC(temp)

DIAGNOSTIC("We now print the curvature/clique score of each node in the

network")

# Now we run over all nodes checking if the node belongs to one clique or

another

# Sc stores the participation rank, which is an earlier step for

computing the curvature

Sc=[]

for node in G.nodes(): # now we process for each clique

score = 0 # This is the initial score of the node in the

participation rank

for clique in temp:

k = len(clique)

if node in clique:

score += 1 # If the node is in the clique raises the score

lista[node][k-1] += (-1)**(k+1)*1/k # Increases the curvature

score for a size k with a different weight due to Gauss-

Bonnet theorem

Sc.append(score)

DIAGNOSTIC("The node " + str(node) + " has score =" + str(score))

total = []

for elements in lista:

total.append(sum(elements)) # This is good if one wants to normalize

by the maximum

DIAGNOSTIC(total)

# nt is normalized by the sum

# nt2 is normalized by the max

nt=[]

nt2=[]

most = np.argsort(-np.array(total))

for i in most:

DIAGNOSTIC("The node " + str(i)+ " is in " + str(total[i]) +

"cliques")

DIAGNOSTIC("These are the most important nodes ranked according to the

total clique score")

DIAGNOSTIC(most)

DIAGNOSTIC("These is the array nt")

  DIAGNOSTIC(nt)

DIAGNOSTIC("These is the array nt2")

  DIAGNOSTIC(nt2)

DIAGNOSTIC("These is the array lista")

DIAGNOSTIC(lista)

DIAGNOSTIC("The output is one vector normalizing the value from the

maximum")

# curv gives the curvature - return Sc instead of curv to get the

particiaption rank - notice that you can normalize this quantity in many

ways

curv=[]

for i in range(0, len(lista)):

curv.append(sum(lista[i]))

curv = np.array(curv)

return curv
